# Supplementary figures and images for: Functional role for Cas cytoplasmic adaptor proteins during cortical axon pathfinding
Source: PLoS Genet. 2025 Nov 7;21(11):e1011941. doi: 10.1371/journal.pgen.1011941 (PMC12611149; doi:10.1371/journal.pgen.1011941)

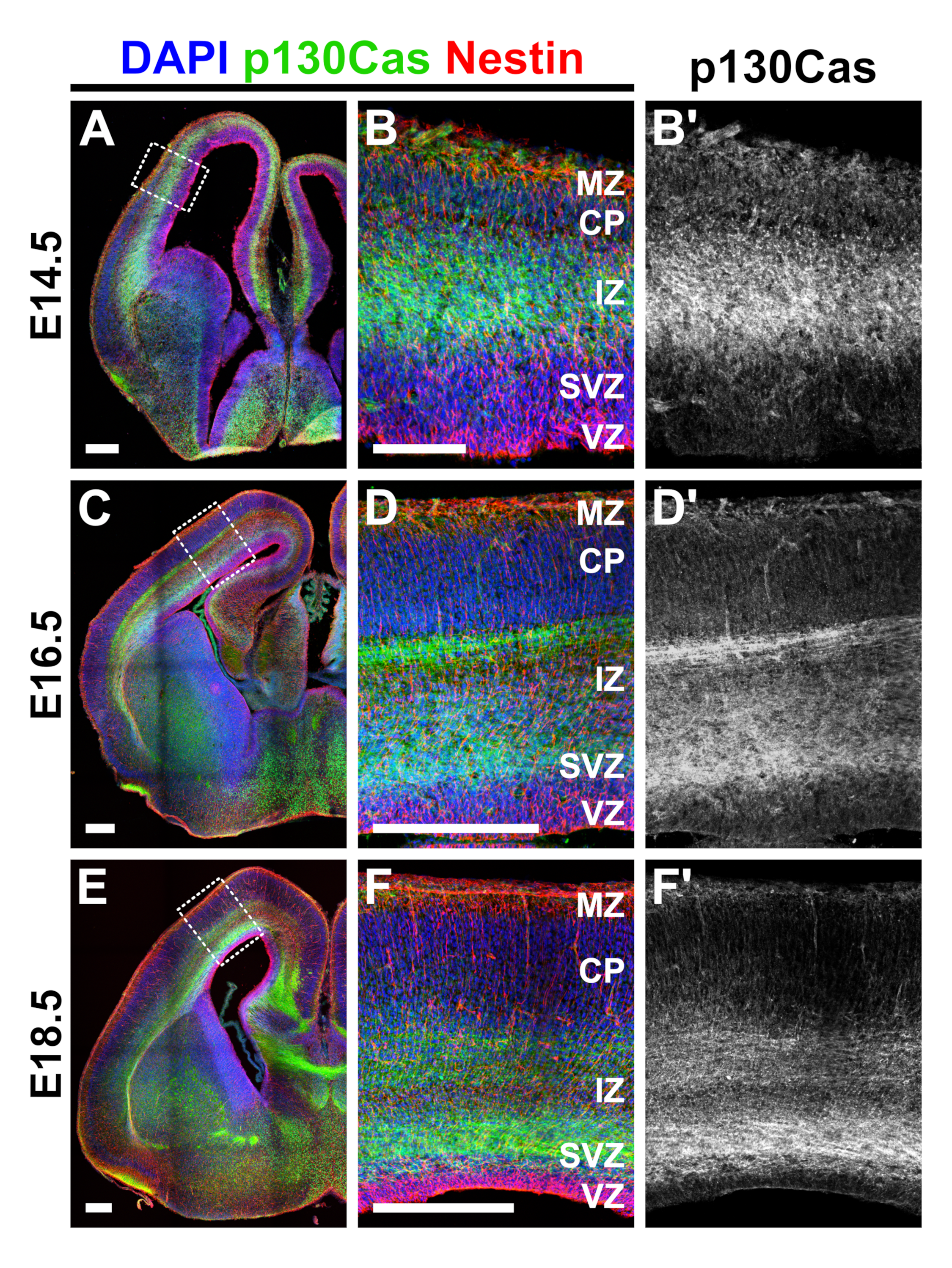

Supplement: S1 Fig — (A-F') Immunohistochemistry showing the developmental expression pattern of p130Cas protein (green) during wild-type cortical development. At E14.5, p130Cas protein expression overlapped with the radial glial cell marker Nestin (red) in the cortical plate and at the pial surface (B-B'). At E16.5, p130Cas expression is strongest in the cortical white matter, subventricular zone, and marginal zone (D-D'). By E18.5, p130Cas is robustly expressed in the subventricular and intermediate zones (F-F'). Weaker expression of p130Cas is also detected at the marginal zone and pial surface at E16.5 (D’) and E18.5 (F'), overlapping with Nestin expression (D and F). MZ = Marginal Zone; CP = Cortical Plate; IZ = Intermediate Zone; SVZ = Subventricular Zone; VZ = Ventricular Zone. Scale bar = 250µm (A, C-F’). Scale bar = 100µm (B-B’). E14.5: n = 4; E16.5: n = 3; and E18.5: n = 4. (TIF) [file pgen.1011941.s002.tif]

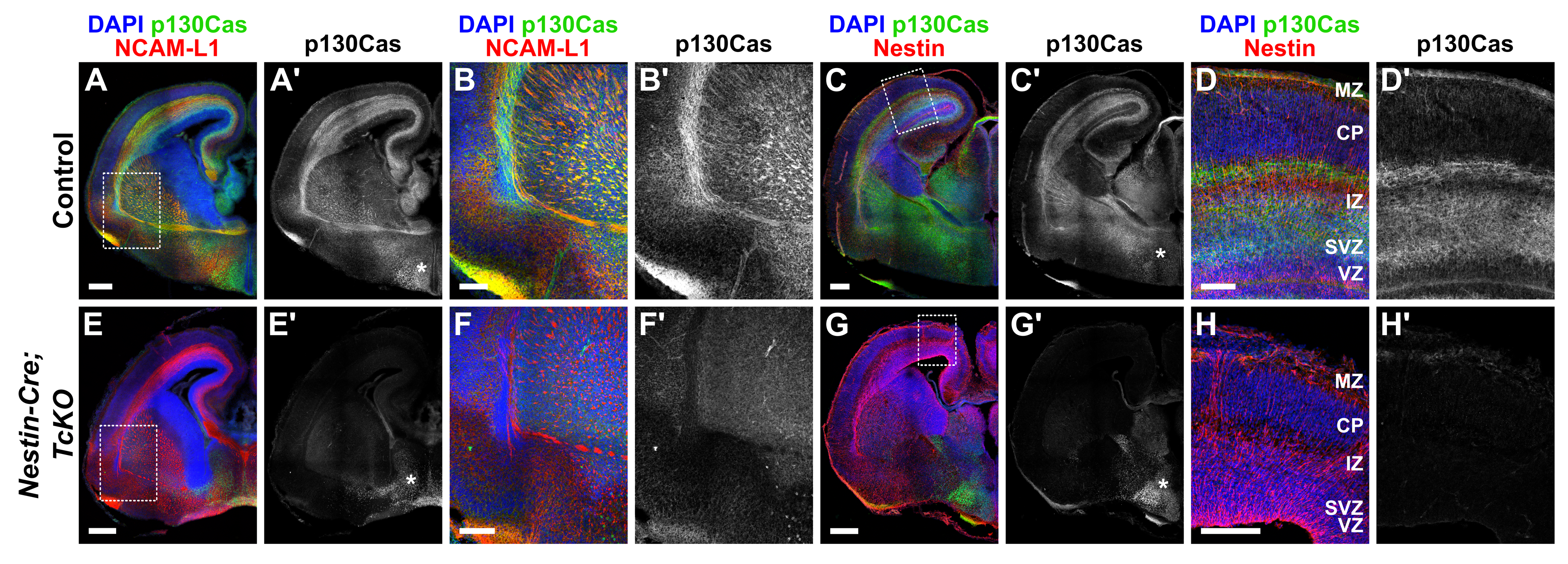

Supplement: S2 Fig — (A-H') Immunohistochemistry of E16.5 Nes-Cre;TcKO and littermate control embryos (CasL-/-;Sin-/-;p130Casfl/+) demonstrates p130Cas signal (green) is not detected in Nes-Cre;TcKO mice. Control embryos strongly express p130Cas protein in the developing neocortex (D') and cortical white matter tracts (A'), including the External Capsule (EC) and Anterior Commissure (AC) (B'). p130Cas signal is lost in Nes-Cre;TcKO embryos (E', F', and H') except for in the hypothalamic region (E' and G', white asterisk). Note that the expression of p130Cas looks nearly identical in control CasL-/-;Sin-/-;p130Casfl/+ embryos and wild-type embryos (Figs 1 and S1). MZ = Marginal Zone; CP = Cortical Plate; IZ = Intermediate Zone; SVZ = Subventricular Zone; VZ = Ventricular Zone. Scale bar = 250µm (A-A', C-C', E-E', and G-G'). Scale bar = 100µm (B-B', D-D', F-F', and H-H'). n = 3 animals for Nes-Cre;TcKO and control. (TIF) [file pgen.1011941.s003.tif]

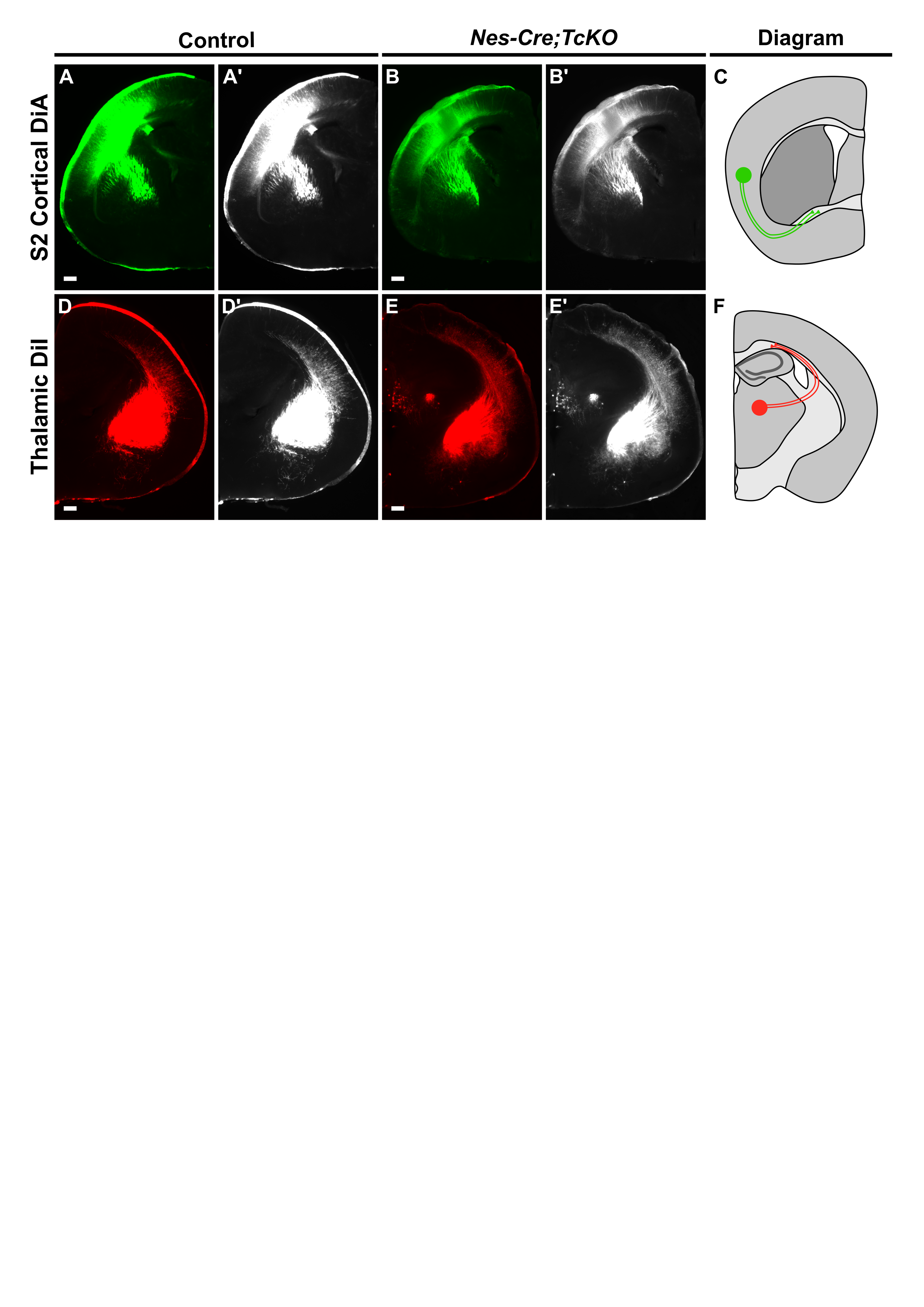

Supplement: S3 Fig — (A-F) DiA and DiI crystal placement in coronal sections from P0 Nes-Cre;TcKO and control mice. DiA crystals were placed into the Somatosensory 2 (S2) region of the cortex (C), while DiI crystals were placed along the central region of the thalamus (F). Scale bar = 250µm. (TIF) [file pgen.1011941.s004.tif]

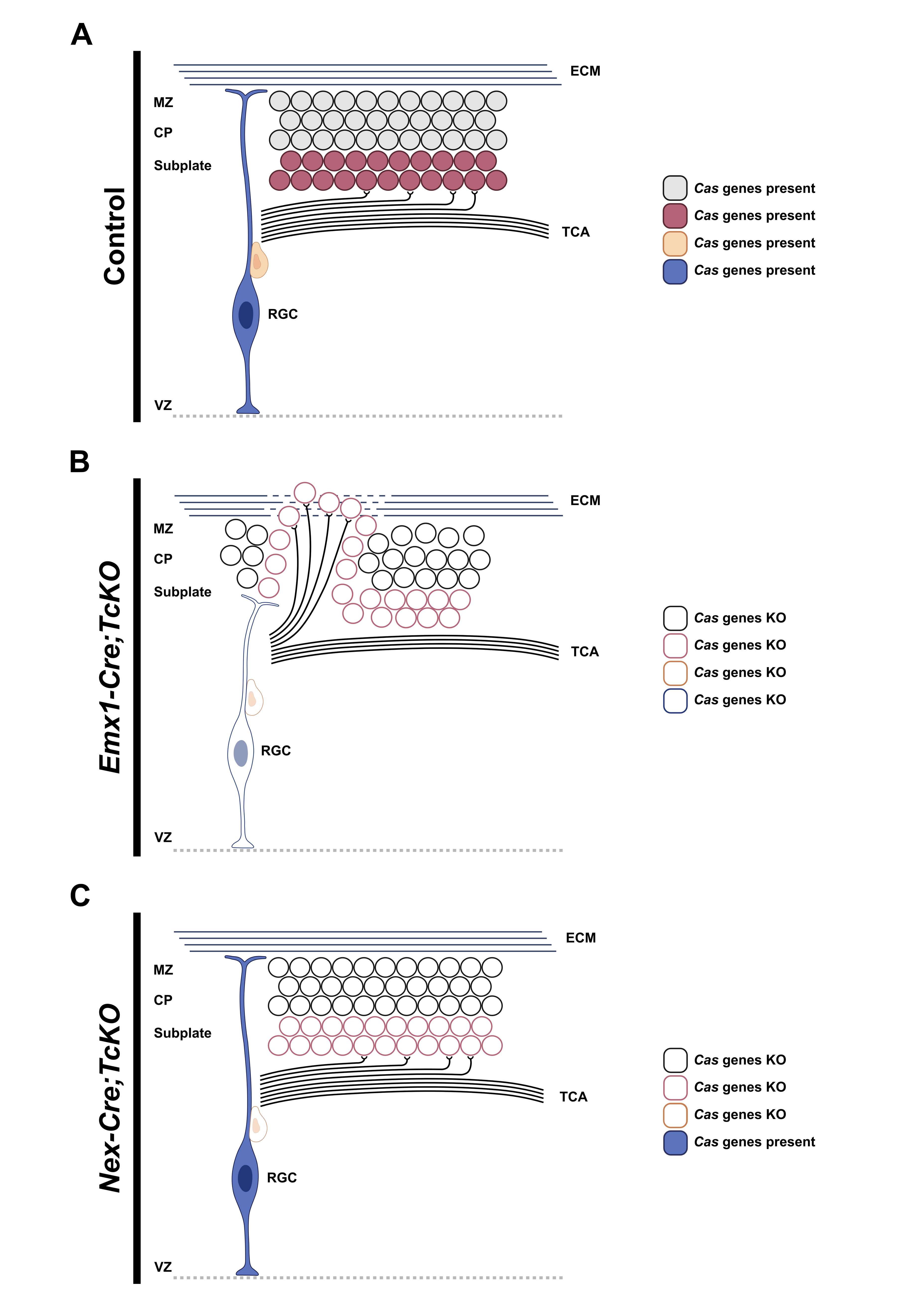

Supplement: S4 Fig — (A-C) Representative model depicting the differences in Cas gene expression in Control, Emx1-Cre;TcKO, and Nex-Cre;TcKO animals. (A) In control animals, Cas genes are expressed in radial glial cells (RGCs; solid blue), postmitotic immature migrating neurons (solid yellow), and post-migratory excitatory cortical neurons (solid grey), including the subplate (solid red). (B) Emx1-Cre drives recombination in cortical RGCs. Thus, in Emx1-Cre;TcKO mutants, Cas genes are ablated in RGCs (hollow blue), immature migrating excitatory neurons (hollow yellow), and excitatory neurons (hollow grey), including the subplate (hollow red). Thalamocortical axons (TCAs) project to mispositioned subplate cells, resulting in the Cortical Bundle (CB) phenotype observed in Emx1-Cre;TcKO mutant mice. (C) In Nex-Cre;TcKO animals, Nex-Cre drives recombination in early postmitotic excitatory cortical neurons. Cas genes are therefore present in RGCs (solid blue), but not in migrating neurons (hollow yellow) and post-migratory neurons of the developing neocortex (hollow grey and hollow red). TCA projections are normal in Nex-Cre;TcKO mutants. ECM = Extracellular Matrix; MZ = Marginal Zone; CP = Cortical Plate; VZ = Ventricular Zone; RGC = Radial Glial Cell; TCA = Thalamocortical Axons. Created with BioRender.com. (TIF) [file pgen.1011941.s005.tif]

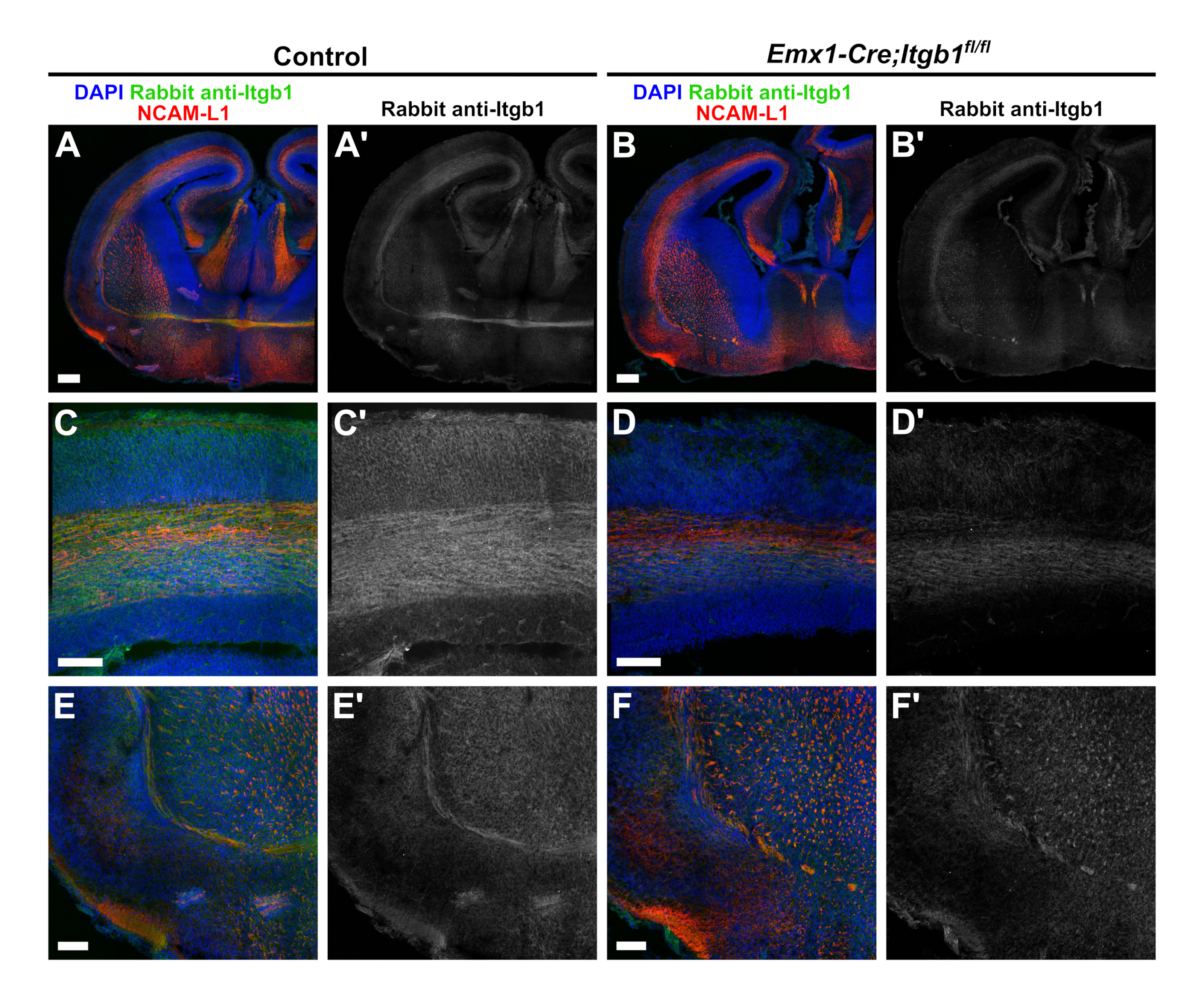

Supplement: S5 Fig — (A-F') Immunohistochemistry of E16.5 Emx1-Cre;Itgb1 and littermate control animals using the rabbit-anti-β1-integrin antibody (Cell Signaling Technology) demonstrates that putative β1-integrin signal (green) is still present in the cortex of Emx1-Cre;Itgb1 mice. Control embryos show signal in the marginal zone (C'), cortical plate, and all major cortical white matter tracts (A'), including thalamocortical axons (C'), the External Capsule (EC) (E'), and the Anterior Commissure (AC) (E'). This signal is still observed in Emx1-Cre;Itgb1 animals, including the cortical plate, EC, and AC (B', D', and F'). Scale bar = 250µm (A-B'). Scale bar = 100µm (C-F'). n = 4 animals for Emx1-Cre;Itgb1 and control. (TIF) [file pgen.1011941.s006.tif]

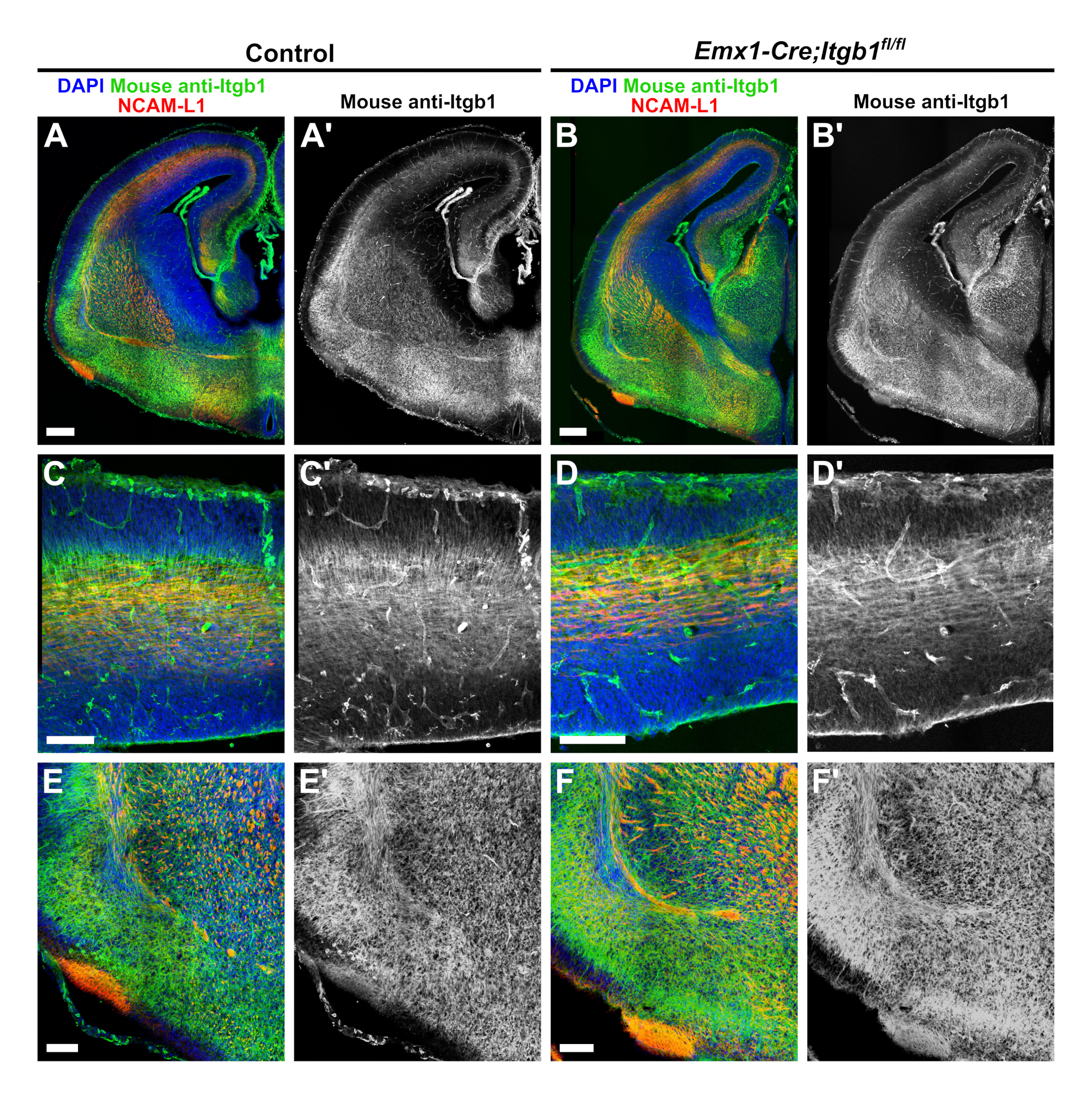

Supplement: S6 Fig — (A-F') Immunohistochemistry of E16.5 Emx1-Cre;Itgb1 and littermate control animals using the mouse-anti-β1-integrin antibody (eBioscience) shows that putative β1-integrin signal (green) is still present in the cortex of Emx1-Cre;Itgb1 mice. Control embryos show signal in the vasculature (C'), the piriform cortex (E'), marginal zone, and all major cortical white matter tracts (A'), including thalamocortical axons (C'), the External Capsule (EC) (E'), and the Anterior Commissure (AC) (E'). Strong signal is still detected in the cortex, AC, and EC of Emx1-Cre;Itgb1 animals (B'). High-magnification images show clear β1-integrin signal in thalamocortical axons (D') and the EC and AC (F'). Scale bar = 250µm (A-B'). Scale bar = 100µm (C-F'). n = 4 animals for Emx1-Cre;Itgb1 and control. (TIF) [file pgen.1011941.s007.tif]

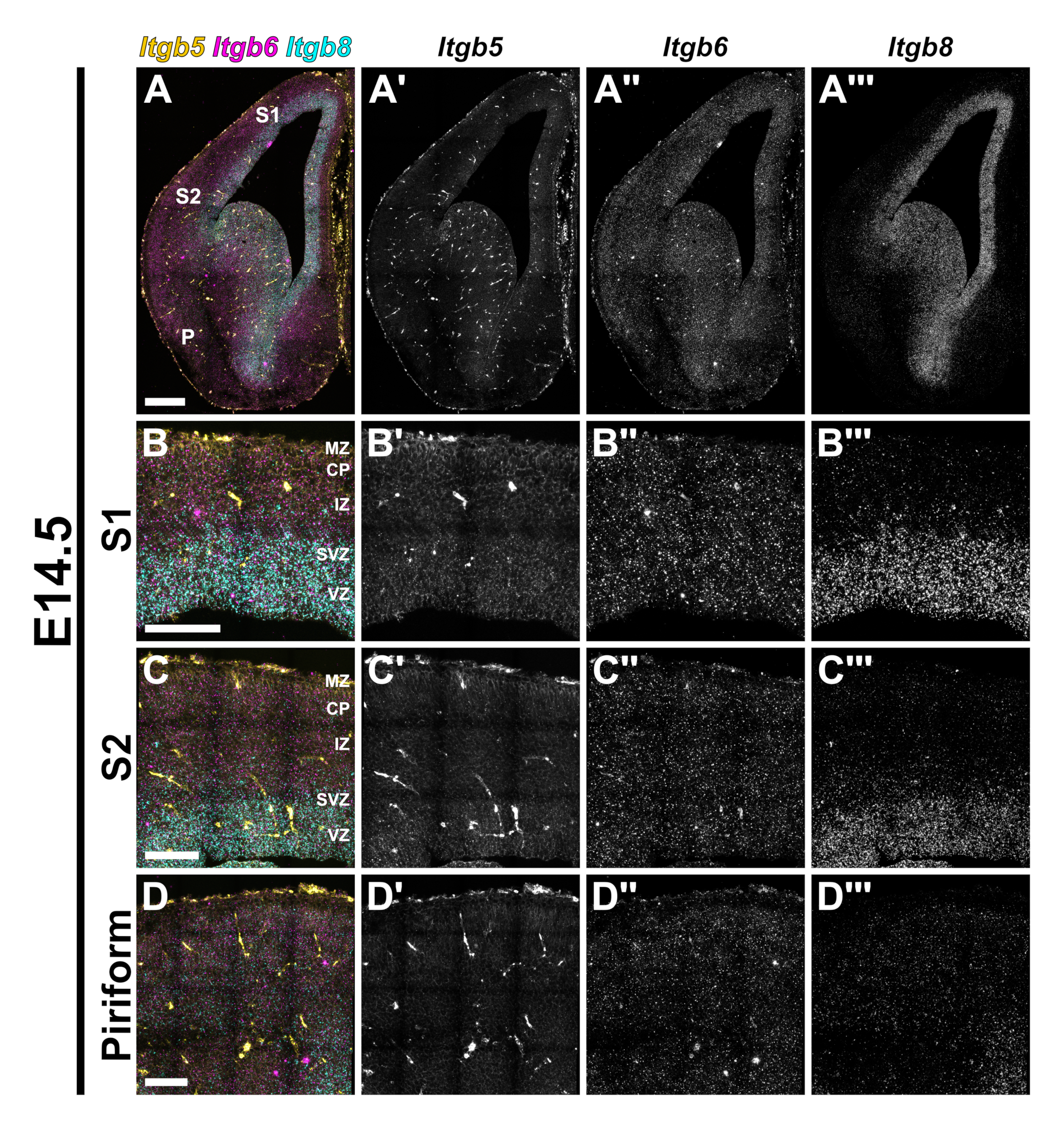

Supplement: S7 Fig — (A-D''') RNAScope for Itgb5 (yellow), Itgb6 (magenta), and Itgb8 (cyan) in the developing neocortex at E14.5. Itgb6 is expressed diffusely throughout the developing neocortex (A'', B'', and C''), while Itgb8 is robustly expressed in the subventricular and ventricular zones of the Primary Somatosensory Cortex (S1) (B''') and Secondary Somatosensory Cortex (S2) (C'''). Itgb5 appears to be weakly expressed in the ventricular zone as well as in the vasculature (A’). High magnification images show diffuse expression of Itgb6 (D'') and Itgb8 (D''') in the Piriform Cortex (P). S1 = Primary Somatosensory Cortex; S2 = Secondary Somatosensory Cortex; P = Piriform Cortex. MZ = Marginal Zone; CP = Cortical Plate; IZ = Intermediate Zone; SVZ = Subventricular Zone; VZ = Ventricular Zone. Scale bar = 250µm (A-A'''). Scale bar = 100µm (B-D'''). n = 3 animals. (TIF) [file pgen.1011941.s008.tif]

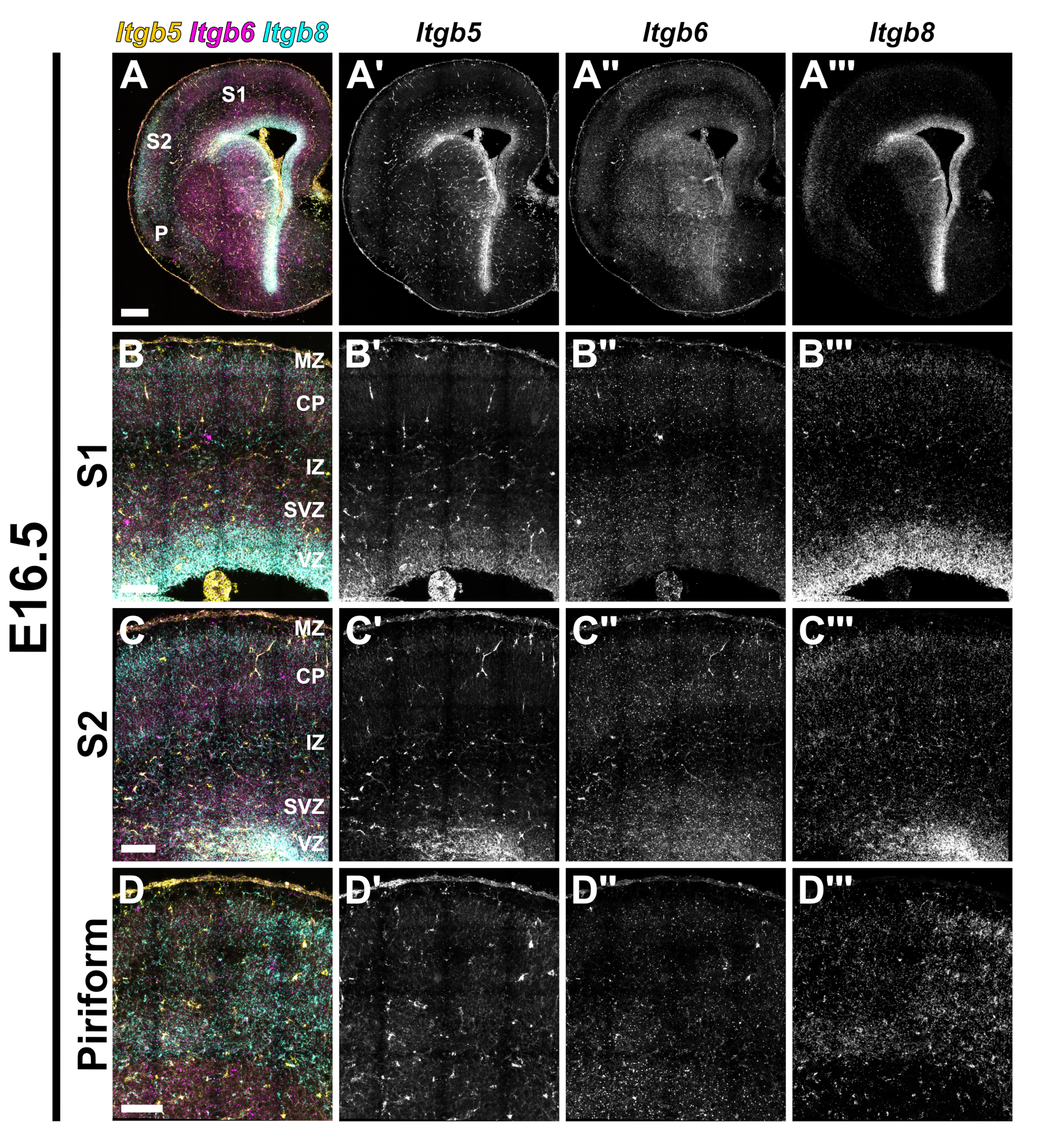

Supplement: S8 Fig — (A-D''') RNAScope for Itgb5 (yellow), Itgb6 (magenta), and Itgb8 (cyan) in the developing neocortex at E16.5. Itgb5, Itgb6, and Itgb8 are all expressed in the ventricular zone of the Primary Somatosensory Cortex (S1) (B'-B''') and Secondary Somatosensory Cortex (S2) (C'-C'''). Itgb5 also appears to be expressed in the vasculature (A'). Weak expression of Itgb6 (B'' and C'') and Itgb8 (B''' and C''') is detected in the marginal zone of S1 and S2. Expression of Itgb6 (D'') and Itgb8 (D''') is observed in the Piriform Cortex (P). S1 = Primary Somatosensory Cortex; S2 = Secondary Somatosensory Cortex; P = Piriform Cortex. MZ = Marginal Zone; CP = Cortical Plate; IZ = Intermediate Zone; SVZ = Subventricular Zone; VZ = Ventricular Zone. Scale bar = 250µm (A-A'''). Scale bar = 100µm (B-D'''). n = 3 animals. (TIF) [file pgen.1011941.s009.tif]

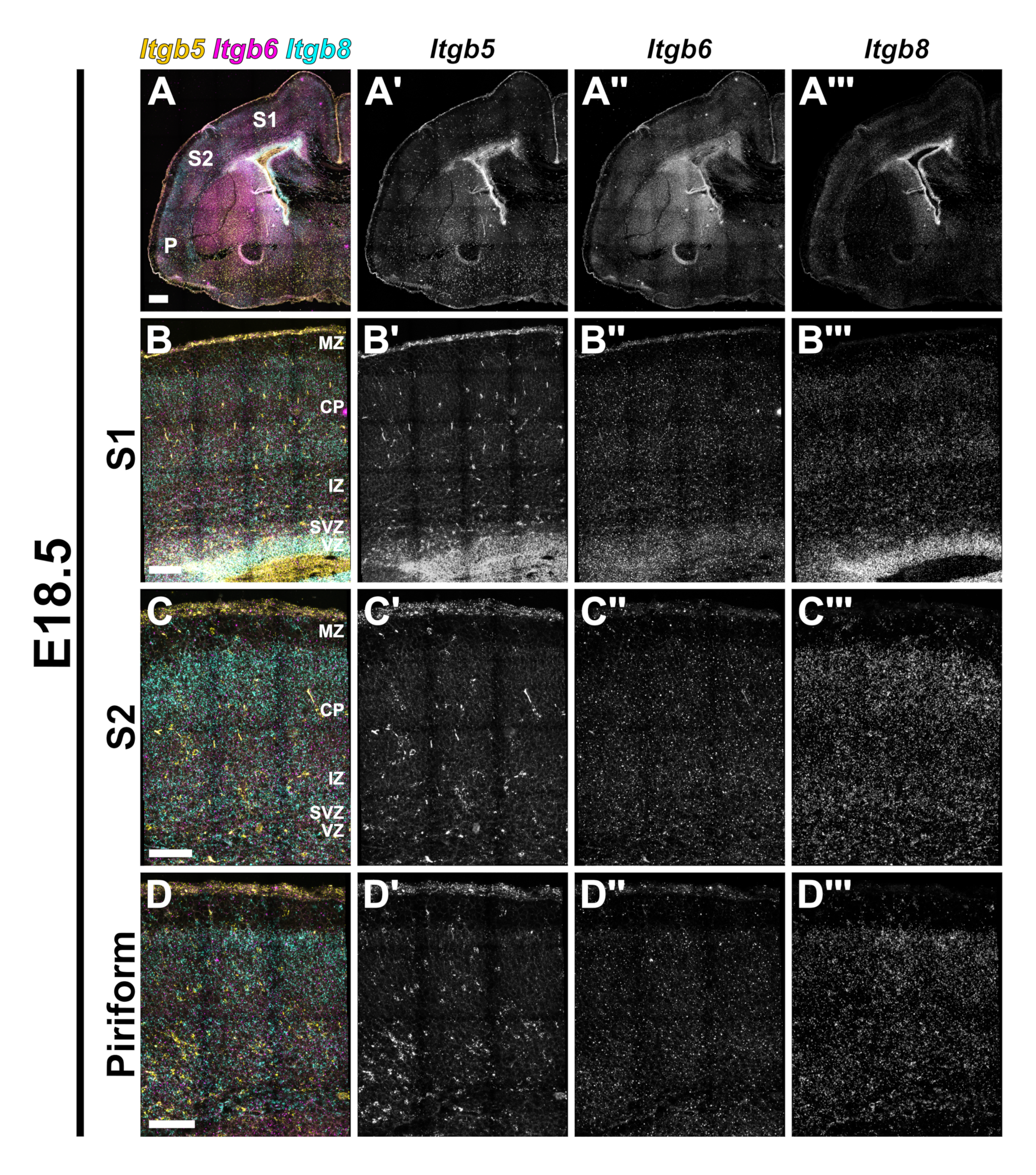

Supplement: S9 Fig — (A-D''') RNAScope for Itgb5 (yellow), Itgb6 (magenta), and Itgb8 (cyan) in the developing neocortex at E18.5. All three transcripts are strongly expressed in the ventricular zone of the Primary Somatosensory Cortex (S1) (B'-B'''). Itgb5 appears to be weakly expressed in the vasculature (B', C', and D'). Itgb8 is diffusely expressed in the Secondary Somatosensory Cortex (S2) (C'''). Expression of Itgb6 (D'') and Itgb8 (D''') is detected in the Piriform Cortex (P). S1 = Primary Somatosensory Cortex; S2 = Secondary Somatosensory Cortex; P = Piriform Cortex. MZ = Marginal Zone; CP = Cortical Plate; IZ = Intermediate Zone; SVZ = Subventricular Zone; VZ = Ventricular Zone. Scale bar = 250µm (A-A'''). Scale bar = 100µm (B-D'''). n = 3 animals. (TIF) [file pgen.1011941.s010.tif]

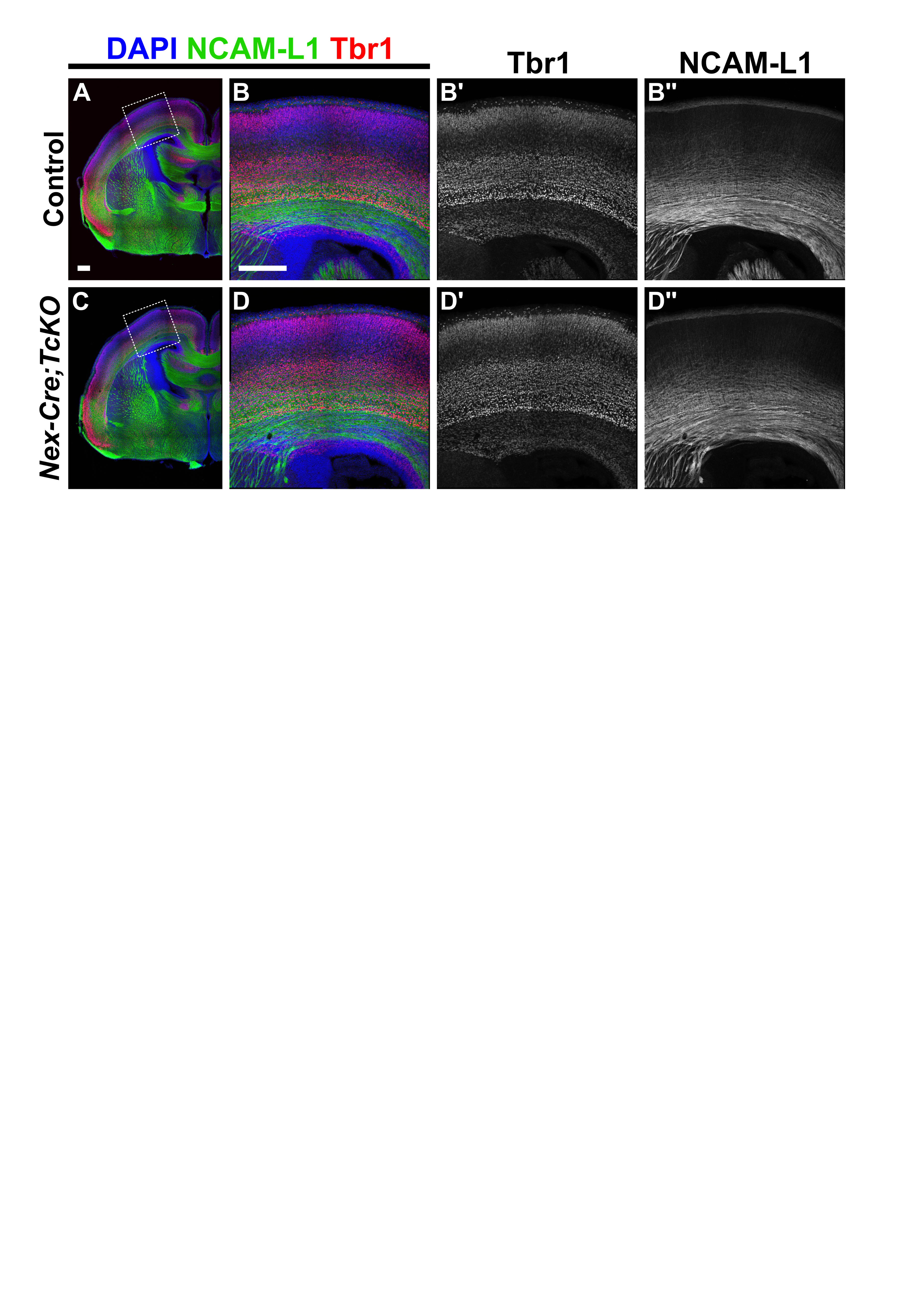

Supplement: S10 Fig — (A-D'') Immunohistochemistry of P0 Nex-Cre;TcKO animals using the axonal marker NCAM-L1 (green) and the deep layer marker Tbr1 (red) shows no apparent differences in lamination between Nex-Cre;TcKO and control cortices. Normal positioning of deep-layer/subplate cells is observed in Nex-Cre;TcKO animals (D'). This correlated with the normal fasciculation and pathfinding of NCAM-L1+ (green) cortical white matter tracts. n = 4 for Nex-Cre;TcKO and controls. Scale bar = 250µm (A and C). Scale bar = 100µm (B-B'', D-D''). (TIF) [file pgen.1011941.s011.tif]
