# Supplementary material for: Functional role for Cas cytoplasmic adaptor proteins during cortical axon pathfinding
Source: PLoS Genet. 2025 Nov 7;21(11):e1011941. doi: 10.1371/journal.pgen.1011941 (PMC12611149; doi:10.1371/journal.pgen.1011941)
Supplement: Attachment — Submitted filename: Response to reviewers-final.pdf [file pgen.1011941.s013.pdf]

Response to reviewers – Plos Genetics

We thank the reviewers for their positive assessment of the revised manuscript and suggestions. We also thank them for taking the previous reviews and our proposed revision plan into consideration. We are pleased that the reviewers found this to be a "well designed and executed study" that "makes a significant contribution to the field," with data that are "of overall high quality," "convincing," and "well-supported by the data." We particularly appreciate the recognition that our work demonstrates "several novel and important findings for the field" through an "elegant and rigorous dissection of Cas function in forebrain axon tract formation" that will be "of specific interest to those studying axon guidance and of broad interest to those studying neural development and circuit formation."

In response to the reviewers' comments from the first round of Review Commons, and following our proposed revision plan, we have completed extensive additional experiments (New **Fig 1** and **S1**, **S2**, **S4**, **S5**, **S6**, **S7**, **S8**, **S**9 and **S10 Figs**) and analyses that significantly strengthen the manuscript:

1. **Quantification of neuronal mispositioning and cortical bundle correlation**: We now provide detailed quantification demonstrating the coordination between misplaced Tbr1+ neurons and cortical bundle formation in *Emx1-Cre;TcKO* and *Emx1-Cre;Itgb1* mutants at E16.5 and P0 (**Figs 8** and **9**). We also present new data showing that Tbr1+ cells are not misplaced in *Nex-Cre;TcKO* mutants (new **S10 Fig**), providing strong evidence for the correlation between misplaced cells and cortical bundle formation.
2. **Protein expression analysis**: We now present immunohistochemical data showing p130Cas protein expression in the developing cortex (new **Fig. 1**, **S1 Fig** and **S2 Fig**). We attempted to detect Itgb1 protein expression using two different antibodies; however, the staining was not specific, as signal remained in the cortex of *Emx1-Cre;Itgb1* mutants (new **S5** and **S6 Figs**). As mentioned in our revision plan, we are limited by the reagents currently available to us.
3. **Improved figure presentation**: We have added boxed regions to denote zoom areas where appropriate throughout the manuscript and provide a schematic diagram illustrating DiI crystal placement for all lipophilic tracing experiments (improved **S3 Fig**).
4. **Alternative β-integrin expression analysis**: We present new RNAscope analysis examining the expression of integrin β5, β6, and β8 in the developing cortex at multiple developmental stages (E14.5, E16.5, and E18.5), revealing expression patterns that may explain why β1-integrin deficiency does not replicate the anterior commissure phenotype observed in Cas mutants (new **S7 Fig**, **S8 Fig** and **S9 Fig**).
5. **Graphical summary and improved discussion**: We have created a graphical summary that clearly illustrates our model of how Cas genes function to guide

TCAs through non-neuronal-autonomous mechanisms (via cortical lamination; new **S4 Fig**).
6. **Enhanced mechanistic discussion**: We have significantly expanded the Discussion to provide a more detailed mechanistic model of how Cas genes function in radial glial cells to regulate cortical lamination, which subsequently affects thalamocortical axon guidance. We have also better articulated the specific novel contributions of our study to the field.

Below are our detailed point-by-point responses to the reviewers' comments from this second round of review at PLOS Genetics.

Reviewers' comments:

<u>**Reviewer #1:**</u>

We sincerely thank Reviewer #1 for their thoughtful and enthusiastic assessment of our manuscript. We are gratified that the reviewer finds our study "well-designed and executed," with "convincing" data, "easy to follow" presentation, and writing that is "well written," demonstrating "several novel and important findings for the field" that will be of interest to those studying axon guidance, neural development, and circuit formation.

We are especially grateful for the reviewer's support regarding the experiments on integrin family members. While the reviewer noted these experiments were "not necessary" to support our main conclusions and would be "outside the scope of the current study," we nevertheless undertook these additional experiments to address the critiques from a previous reviewer (#2 from Review Commons)

Finally, we were happy to address the suggested “very minor revisions” to further strengthen the manuscript.

1) I fully agree with Reviewer #1 from the Review Commons reviews that the terminology of cortical-autonomous and non-neuronal autonomous is challenging. At best it is cumbersome, at worst it is sometimes inaccurate. I highly recommend finding new wording to describe these interpretations. A good example is in the paragraph comprising lines 267-274. The sentence "The proper fasciculation of AC axons appears to require Cas in a neuron-autonomous manner, likely reflecting a requirement for Cas genes in the projecting axons themselves." The second part of the sentence is much clearer than the first half. "Neuronal-autonomous" is unnecessary and can be confusing, especially since the authors point out that the mutant axons might be erroneously following non-mutant WT axons. Continuing in that paragraph: "In contrast, the proper projection of TCA afferent into the cortex appears to have a non-neuronal-autonomous requirement for Cas genes. As such, we infer that Cas genes are required by some

non-neuronal cortical cell population..." The second sentence is much more effective in conveying the message than the first sentence. "Non-neuronal-autonomous" is not a phrase that is used often in the field, and is likely to confuse many people. It would be much clearer to say something like "required by a non-neuronal population, but not by neurons themselves." Also, to say that the bundling phenotype is "non-neuronal-autonomous" can be understood as inaccurate, since the displaced neurons are likely causing the bundling phenotype. It's true that the Cas genes seem to be required in the progenitors and not in the neurons, but the neurons are definitely involved. Because of the high likelihood that readers will be confused by these terms, I highly recommend completely removing the terms "cortical autonomous", "neuronal-autonomous", and non-neuronal-autonomous" and just using different words to describe these ideas.

We fully agree with Reviewer #1 and the previous Reviewer #1 from Review Commons that the terminology of "cortical-autonomous," "neuronal-autonomous," and "non-neuronal-autonomous" is challenging and potentially confusing. We appreciate the specific examples provided, particularly the observation that phrases like "required by a non-neuronal population, but not by neurons themselves" are much clearer than "non-neuronal-autonomous." We also agree that "non-neuronal-autonomous" is not commonly used in the field and that the displaced neurons are indeed involved in the bundling phenotype, even though Cas genes appear to be required in progenitors rather than neurons themselves.

We have addressed this critique by implementing several changes throughout the manuscript:

1. **We have removed or substantially reduced the use of the terms "cortical-autonomous," "neuronal-autonomous," and "non-neuronal-autonomous" throughout the paper, replacing them with clearer, more direct language as suggested by the reviewer. For example, we now define requirements more explicitly (e.g., "requiring Cas gene function in the developing neocortex itself" or "required by a non-neuronal cortical cell population").**
2. **We now provide a graphical abstract to visually clarify the requirement for Cas genes in different cell populations.**
3. **We have added clarifying sentences to the previous Lines 239-247 (now Lines [289-296]) to better explain these concepts using more straightforward language.**

We believe these changes significantly improve the clarity and accessibility of our manuscript.

2) Line 254: "(i.e. requiring Cas gene function in the cortical plate itself)" is not accurate. The interpretation is that Cas genes are required in the developing cortex (or developing

neocortex or developing dorsal forebrain, etc), but not necessarily in the cortical PLATE (which refers to the postmitotic neurons that are not in the VZ/SVZ, IZ, MZ).

We thank the reviewer for catching this oversight. We agree that "cortical plate" was inaccurate as it specifically refers to postmitotic neurons, and have now changed the wording to "developing neocortex" throughout the manuscript.

3) Line 290: Sentence ending with "n=3 for controls)" is missing a period at the end.

A period has been added at the end of this sentence.

4) The term "interpolate" (e.g., lines 292, 305, etc) is a bit unusual. Consider changing to "intercalate". I understand they can mean the same thing, but it seems "intercalate" is used more frequently to describe axons and "interpolate" is used more frequently in the mathematical sense. However, if this is the preferred term in the field, feel free to ignore.

We have changed "interpolate" to "intercalate" throughout the manuscript, as the latter is indeed more commonly used to describe axon behavior in the field.

5) Lines 350-351: the phrase "(Figure 7 and Figure 8, respectively)" is in an unusual place in the sentence, making it unclear whether Figure 7 and Figure 8 are split based on ages (E16.5 vs P0), mutant genotype (TcKO vs Itgb1), or markers (NCAM-L1 vs Tbr1).

We have changed the phrasing to: "Thus, we performed immunohistochemistry in *Emx1-Cre;TcKO* and *Emx1-Cre;Itgb1$^{fl/fl}$* mutants at E16.5 (Figure 8) and P0 (Figure 9), using the markers NCAM-L1 and Tbr1."

6) This is not a major point but may be worth thinking about and possibly discussing: The number of stray axons in mutant brains is always bimodal, with some around 5 and others around 10 - is this meaningful or interesting? The exception is when using the Ai14 reporter, where all are at 10 - is there a reason for this? In your other brains where the mutant axons are not traced, are you missing some stray axons to get the average of 5 in some brains?

We appreciate the reviewer raising this interesting observation. We also noticed this bimodal distribution primarily in Emx1Cre;TcKOs, though we do not observe the same trend around these specific numbers in other genotypes. At this time, we are uncertain whether this represents a meaningful biological feature or an artifact of our somewhat reduced sample size. We agree this is an intriguing

pattern that warrants further investigation in future studies, but we do not currently have sufficient data to draw definitive conclusions about its significance.

Reviewer #2:

We sincerely thank the reviewer for their thoughtful assessment of our manuscript and revisions. We are pleased that the reviewer finds our data "generally of high quality" and agrees that our "findings presented are supported by the data." We particularly appreciate the reviewer's recognition that we have adequately addressed the concerns raised in the previous Review Commons reviews and that our "changes made, or planned changes, will be sufficient to produce a rigorous manuscript." We are grateful for the additional minor comments provided to further strengthen our work, and we address each of these points below.

1. The authors pursue a genetic strategy which will delete three of the four Cas proteins. There is no mention of how these three are chosen or why Cass4 is omitted. I am not suggesting a quadruple knockout is necessary but the rationale may be useful to share.

We have now added text to the Introduction clarifying the rationale for targeting these three specific Cas family members. As we now note in the manuscript: "While p130Cas (Bouton et al. 2001), CasL/Nedd9 (Aquino et al. 2008), and Sin/Efs (Ishino et al. 1997) are all expressed in the embryonic nervous system, Cass4 expression appears to be restricted primarily to lung, spleen, and blood cells with minimal brain expression (Singh et al. 2008)." We also added this sentence a few lines below: "Accordingly, we focused our investigation on the three Cas family members with established expression and functional roles in neural development."

2. Fewer non-standard abbreviations would likely help readers not fully immersed in the field. These include PSPB, DTB, a/pAC, CB.

We thank the reviewer for this helpful suggestion to improve accessibility for readers. We have made the following changes:

1. **We now spell out PSPB (pallial-subpallial boundary) and DTB (diencephalon-telencephalon boundary) throughout the manuscript to improve clarity.**

2. **We have also ensured that abbreviations are defined in each section. We respectfully chose to retain the abbreviations a/pAC (anterior/posterior limb of the anterior commissure) and CB (cortical bundles). The a/pAC abbreviations are commonly used in the anterior commissure literature, and both terms are mentioned multiple times throughout the manuscript. We believe that spelling out these longer terms repeatedly would make the text more cumbersome to read, but we have ensured they are clearly defined at first use in each relevant section.**

3. The colocalization in the cortical plate at E14.5 in Fig 1 (p130Cas & NCAM) is not apparent. Other data points are convincing.

We believe the image is representative of what we observe at this developmental stage. The external capsule (EC) is indeed quite thin at E14.5, which may make the colocalization less immediately apparent. However, upon careful examination of both the merged image and the individual black and white channels, there is clear regional overlap between the p130Cas and NCAM signals within the EC. We have confirmed this colocalization pattern across multiple sections and embryos. To improve clarity, we have changed the way we mark the EC in the figure, now using arrows to make the region more readily identifiable for readers. Additionally, we have revised the text to describe this as "overlap" rather than "colocalization" to more accurately reflect the nature of the observed signal distribution at this early developmental stage.

4. The authors use the common convention of abbreviating p-values with annotations like ****. I think a better and emerging style is to show the actual p-value and to let the reader make their own conclusions about statistical “significance.”

We appreciate the reviewer's perspective on p-value reporting. We use the asterisk notation in the figures for visual simplicity and ease of interpretation. However, we report the actual p-values in the text of the Results section, allowing readers to evaluate the statistical evidence in detail. We believe this approach balances visual clarity in the figures with the precision and transparency that readers need to draw their own conclusions about the data.

5. In figure 4b’, the Emx1-Cre defasciculation is a bit hard to see.

We thank the reviewer for this observation. To improve visualization of the defasciculation phenotype, we have replaced the selected images Figure 4b-b’

(now Figure 5b-b') with images from another animal that more clearly presents the defasciculation phenotype. The same imaging parameters were used for all images for all genotypes to ensure fair comparison.

6. I personally do not prefer A, A', A'' vs. A,B,C in labeling figures but that is a matter of personal opinion.

We appreciate the reviewer sharing their perspective on figure labeling conventions. We recognize that both A, B, C and A, A', A'' nomenclatures are used in the literature, and while we have used the A, B, C format in other contexts, we have opted to use the A, A', A'' format in this manuscript. We chose this convention because the primed panels represent higher magnification views or alternative visualizations of the same experimental condition shown in the unprimed panels, and we felt this notation best conveys the relationship between the panels.

7. Lines 320-335: can this be reorganized to tell the story in the same logical order used elsewhere in the manuscript?

This is an excellent suggestion. We have reorganized lines 320-335 to follow the same logical order used elsewhere in the manuscript, which we agree improves the flow and readability of this section.

8. The graphical abstract suggested in Review Commons would indeed be a nice addition.

We agree with the reviewer that a graphical abstract would be a valuable addition to the manuscript. We have now created a graphical model that summarizes the key findings and cell-type requirements for Cas genes in cortical axon development.

9. The values plotted are the averages from each animal. This should be explicitly stated in the methods.

We have now added the following text to the Methods section to explicitly clarify this point: "Bar graphs represent the group mean ± standard error of the group mean, with plotted values representing the mean from each animal analyzed."

Reviewer #3:

We sincerely thank the reviewer for their thoughtful assessment of our manuscript and for recognizing that our study "uncovers novel functions for Cas family genes in forebrain axon organization" through an "elegant and rigorous dissection of Cas function in forebrain axon tract formation through mouse genetics" that "makes a significant contribution to the field." We are pleased that the reviewer finds our data to be "of overall high quality" with "rigorous" analyses and conclusions that are "well-supported by the data."

In their response to previous reviews, the authors highlight four drivers of novelty/significance that could be fleshed out in a more detailed discussion section. However, I do not agree that challenging "the assumption that Cas proteins always function downstream of beta1-Integrin" is all that novel, as multiple other ligand-receptor systems have been implicated upstream of Cas activation in neural circuit development.

We appreciate the reviewer's constructive feedback regarding our framing of novelty in our response to the previous round of reviews. The reviewer makes an excellent point that multiple ligand-receptor systems have been implicated upstream of Cas activation in neural circuit development, and we agree that our statement about "challenging the assumption that Cas proteins always function downstream of beta1-Integrin" overstated the novelty of this aspect. In the revised Discussion, we now appropriately highlight the key contributions of our study without overstating their novelty, focusing on what our genetic dissection uniquely reveals about how Cas proteins coordinate distinct mechanisms of forebrain axon organization through both direct and indirect pathways.

These pathways are mentioned multiple times in the manuscript and could be the focus of extended discussion, instead of listing many knockout mouse lines that exhibit anterior commissure defects, even if they do not resemble the Cas knockouts and there is no evidence implicating the relevant molecules upstream of Cas proteins.

We agree with the reviewer that an extensive list of mutant mouse lines with anterior commissure defects is not particularly informative, especially when those phenotypes do not closely resemble the Cas mutants and there is no direct evidence linking those molecules to Cas signaling. We have revised the Discussion to significantly condense this list, retaining only key references to similar genetic studies for context. We have refrained from expanding the

discussion of these various pathways, as we do not have experimental evidence connecting them to Cas function in this process. Instead, we focus the Discussion on the mechanistic insights our genetic dissection provides and the documented upstream regulators of Cas signaling that we mention elsewhere in the manuscript.
