# Supplementary material for: Functional role for Cas cytoplasmic adaptor proteins during cortical axon pathfinding
Source: PLoS Genet. 2025 Nov 7;21(11):e1011941. doi: 10.1371/journal.pgen.1011941 (PMC12611149; doi:10.1371/journal.pgen.1011941)
Supplement: Attachment — Submitted filename: 157066_1_rebuttal_3412226_sxrn9d.docx [file pgen.1011941.s012.docx]

**Manuscript number:** RC-2025-02988

**Corresponding author(s):** Martin Riccomagno

*[The “revision plan” should delineate the revisions that authors intend to carry out in response to the points raised by the referees. It also provides the authors with the opportunity to explain their view of the paper and of the referee reports.*

*The document is important for the editors of affiliate journals when they make a first decision on the transferred manuscript. It will also be useful to readers of the reprint and help them to obtain a balanced view of the paper.*

*If you wish to submit a full revision, please use our "*[*Full Revision*](https://reviewcommons.org/templates/full-revision.docx)*" template.* ***It is important to use the appropriate template to clearly inform the editors of your intentions.****]*

## 1. General Statements [optional]

We thank all three reviewers for their thoughtful and constructive comments. We are pleased that the reviewers found our work to be "very interesting," "well written," with "high quality" data that is "convincing" and will be "of broad interest for the community of axon guidance, circuit formation and brain development." We particularly appreciate the recognition that our study provides "novel functions for Cas family genes in forebrain axon organization" and uses "state-of-the art mouse genetics" with "quantitative and statistical rigor." Below are our detailed responses to each reviewer's comments, including extensive additional experiments and analyses that we will perform to significantly strengthen the manuscript.

## 2. Description of the planned revisions

**Reviewer #1**

**We thank this reviewer for recognizing that our experiments are "carefully done and quantified" with "clear and striking" phenotypes that "support most of the conclusions in the manuscript." We appreciate their acknowledgment that this work will be "of interest to developmental neurobiologists and the axon guidance and adhesion fields."**

**Major Comments:**

**1. Authors clearly show that misplaced TCA axons are coordinate with cortical layer defects, with misplaced tbr1+ neurons, in EMX-Cre cas and integrin knockouts, suggesting these axons are following misplaced cells. These results are described as 100% coordinate, but since there is no figure of quantification, authors need to clarify how many embryos were examined for each genotype, as this was not described in results or legends.**

We apologize for this oversight and will provide detailed quantification of this important finding. We examined a total of 11 *Emx1Cre;TcKO* embryos with 13 controls, and 14 *Emx1Cre;Itgb1* embryos with 13 littermate controls at two developmental stages (E16.5 and P0) to quantify the coordination between misplaced Tbr1+ neurons and cortical bundle formation. This quantification will be presented in the main text and figure legend.

Here's a more detailed breakdown of those numbers: For *Emx1Cre;TcKO* knockouts, we examined 7 controls and 5 mutants at P0, and 6 controls and 6 mutant embryos at E16.5. For the *Emx1Cre;Itgb1* knockouts, we examined 5 controls and 6 mutant neonates at P0, and 8 controls and 8 mutant embryos at E16.5.

**2. Are the neurons not misplaced in Nex cre cas or integrin knockouts? One would think presumably not, but then what are the tbr1+ cell migration defect caused by? I struggle with the semantics of non-neuronal autonomous role of cas in cortex, since tbr1+ neurons are misplaced, and this is what the axons are mistargeting too. So yes, potentially cas or b1 is not needed in those neurons, but those misplaced neurons are presumably driving the phenotype.**

We agree that this important point requires better explanation. You are absolutely correct that Tbr1+ neurons are not misplaced in Nex-Cre;TcKO mutants (Wong et al., 2023), which is precisely why these animals do not exhibit cortical bundle formation. In addition to our previously published data showing normal location of Tbr1+ neurons in those mutants, we can also provide similar analysis at E16.5 and P0 as a supplemental figure. The model we propose is that Cas genes are required in radial glial cells for proper positioning of deep layer cortical neurons. These correctly positioned neurons, in turn, provide appropriate guidance cues for TCA projections. Hence, our model is that while the role of *Cas* genes is non-neuronal-autonomous (acting in radial glia rather than in the neurons themselves), the mispositioned Tbr1+ neurons in *Emx1-Cre;TcKO* mutants drive the TCA misprojection phenotype. We will clarify this mechanism in the discussion and provide a new graphical model as a supplemental figure to facilitate conceptualization of our conclusions.

**3. Authors need to clarify in the discussion that they can't rule out the cas not also needed in tca neurons, Since neither emx or nex cre would hit those cells.**

We will add the following clarification to the discussion: The analysis of cortical bundle formation in *Emx1-Cre;TcKO* revealed a comparable phenotype to that observed in *Nestin-Cre;TcKO,* strongly suggesting a cortical-autonomous role for *Cas* genes in CB formation. “However, we cannot formally exclude a thalamus-autonomous role for *Itgb1* or Cas genes in TCA pathfinding, as we did not ablate these genes exclusively in the thalamus. Future studies using thalamus-specific Cre drivers would be needed to definitively address this question."

**4. Could authors add boxes in zoomed out brain images to denote zoom regions. And potentially a schematic demonstrating placement of DiI for lipophilic tracing experiments.**

We will add boxes to denote zoom regions where possible throughout the manuscript. For some high magnification panels, we selected the best representative images, which don't necessarily correspond to specific regions of the lower magnification panels, but we will note this in the figure legends. We will also add a schematic diagram to a supplemental figure illustrating DiI placement for all lipophilic tracing experiments.

**Reviewer #2**

**We thank this reviewer for describing our study as "very interesting," "well written," with data that are "of high quality" and findings that are "convincing." We appreciate their recognition that we used "state-of-the art mouse genetics" and that our work will be "of broad interest for the community of axon guidance, circuit formation and brain development."**

**Major Comments:**

**1. Immunofluorescence labeling for other β-integrin family members to examine expression in AC axons may provide insights into why β1-integrin deficiency does not replicate the Cas TcKO phenotype.**

This is an excellent suggestion that we will address experimentally. We will perform RNAscope analysis for integrin β5, β6, and β8 in developing piriform and S1 cortex at E14.5, E16.5, and E18.5, as these are the only other β-integrins expressed during cortical development. We anticipate that this analysis may reveal expression of alternative β-integrins in the neurons that extend axons along the developing anterior commissure, which would provide a potential explanation for why β1-integrin deficiency does not replicate the AC phenotype observed in Cas TcKO animals. These new data will be presented as part of a new figure.

**2. Is there any evidence that β1-integrin in developing cortical axons is colocalized with Cas proteins (in vivo or in vitro)?**

We have tested multiple antibodies for p130Cas and CasL without success in cortical tissue. However, we will test two new integrin β1 antibodies and a new p130Cas antibody. While direct colocalization may be challenging due to species restrictions and tissue-specific antibody performance, we will attempt to show regional co-expression in consecutive sections. If the integrin antibodies work, we will present data as a supplemental figure demonstrating that p130Cas (using our BAC-EGFP reporter) and β1-integrin show overlapping expression patterns in developing cortical white matter tracts and neurons, supporting their potential functional interaction. In the end, while we will try to address this critique, we will be limited by the reagents that are available to us.

**Minor Comments:**

**3. How long do the Cas TcKO with the various cre driver survive?**

We have not systematically quantified survival beyond 6 months, but surprisingly, survival up to 6 months of age appears normal for all genotypes examined. This information will be included in the Methods section.

**Reviewer #3**

**We thank this reviewer for acknowledging that our "main claims and conclusions are solidly supported by the data" with "good overall data quality" and "high quantitative and statistical rigor." We appreciate their recognition that we "uncover novel functions for Cas family genes in forebrain axon organization" and that our "overall reporting and discussion of findings is data-driven and refrains from excessive speculation."**

**Addressing Concerns About Novelty and Impact:**

We respectfully disagree with the characterization of our findings as "somewhat incremental." While we acknowledge that similar axonal defects have been described in other lamination mutants, our study makes several **novel and significant contributions**:

1. **First demonstration of Cas family requirement in forebrain axon tract development**: This is the first study to establish roles for Cas proteins in axon guidance, representing a completely new function for these well-studied signaling molecules.
2. **Novel β1-integrin-independent role for Cas proteins**: Our finding that AC defects occur in Cas mutants but not β1-integrin mutants reveals a previously unknown signaling pathway and challenges the assumption that Cas proteins always function downstream of β1-integrin.
3. **Mechanistic insights into cortical-TCA interactions**: While the general principle that cortical lamination affects TCA projections has been established, our work provides the first demonstration of how specific adhesion signaling molecules (Cas proteins) control this process through radial glial function.
4. **Cell-type specific requirements**: Our systematic analysis using multiple Cre drivers provides unprecedented detail about where and when Cas proteins function during brain development, revealing both neuronal-autonomous (AC) and non-neuronal autonomous (TCA) roles.

As Reviewer #2 noted, "The main advancement is a more nuanced understanding of where and when these molecules function during brain development and insights into the origin of the defects observed." This represents significant mechanistic progress in understanding forebrain circuit assembly.

**Cross-Reviewer Comments:**

We appreciate Reviewer #3's agreement with the other reviewers' suggestions and will address the quantification of neuronal mispositioning/axon bundle correlation as requested by Reviewer #1.

## 3. Description of the revisions that have already been incorporated in the transferred manuscript

While we embark on the additional experimental analysis and work on editing and expanding the figures, we have already completed some minor revisions to the text:

1) We now include the quantifications requested by Reviewer 1 (critique #1), also mentioned by Reviewer 3.
2) We have expanded the discussion to address the two other points raised by R1 (critiques #2 and 3).
a) Regarding the possibility of a thalamus-autonomous contribution, we now state: "However, we cannot formally exclude a thalamus-autonomous role for Itgb1 or Cas genes in TCA pathfinding, as we did not ablate these genes exclusively in the thalamus. Future studies using thalamus-specific Cre drivers would be needed to definitively address this question."
b) Regarding expanding the explanation of our model: "Within the context of our previous work (Wong et al. 2023), these results suggest a model whereby Cas genes are required in cortical radial glial cells to regulate endfeet attachment, stabilization of the basement membrane, and glial process organization, which subsequently aid in neuronal migration and formation of the cortical layers, including the subplate. In the absence of Cas or Itgb1 genes, these radial glial tracks are disrupted, resulting in mispositioned subplate neurons (**Figure 7 D and H**, **Figure 8 D and H**; Wong et al. 2023). These mispositioned subplate neurons could potentially recruit TCAs for innervation, causing ectopic cortical bundles to innervate the proper intermediate target positioned in the wrong location. Hence, our model is that while the role of Cas genes is non-neuronal-autonomous (acting in radial glia rather than in the neurons themselves), the mispositioned Tbr1+ neurons in Emx1-Cre;TcKO mutants drive the TCA misprojection phenotype. These results, however indirect, add to an extensive body of literature suggesting that proper lamination and formation of the subplate is required for appropriate TCA afferent projection (McConnell et al. 1989; Ghosh et al. 1990; Ghosh and Shatz 1992; Allendoerfer and Shatz 1994; O'Leary et al. 1994; Molnár et al. 1998a, 2012; López-Bendito and Molnár 2003; Kanold et al. 2003; Kanold 2009; Kanold and Luhmann 2010; Hoerder-Suabedissen and Molnár 2012; López-Bendito 2018; Ohtaka-Maruyama 2020; Molnár and Kwan 2024)."

## 4. Description of analyses that authors prefer not to carry out

**We believe we will be able to address all the major points with new experiments or providing additional clarification. However, there were a couple of minor suggestions that, while very interesting, were noted as optional by the reviewers and are beyond the scope of this study.**

**Suggestion about cell autonomy testing**: We appreciate the optional suggestion to test strict cell autonomy using sparse deletion approaches. While this would indeed be interesting, it would represent a substantial undertaking beyond the scope of the current study. However, we believe our current data using NexCre (which hits early postmitotic neurons) versus NestinCre (CNS-wide deletion) and Emx1Cre (cortical progenitors) provides supportive evidence for neuronal autonomy of the AC phenotype, as mentioned by the reviewer.

**In vitro axon guidance assays**: This is an excellent suggestion for future molecular studies. In the discussion we identify specific candidate guidance molecules (e.g. Ephrins) that would be prime targets for such experiments.
